# Clinical and Cost-Effectiveness of Telehealth-Supported Home Oxygen Therapy on Adherence, Hospital Readmission, and Health-Related Quality of Life in Patients With Chronic Obstructive Pulmonary Disease: Systematic Review and Meta-Analysis of Randomized Controlled Trials

**DOI:** 10.2196/73010

**Published:** 2025-07-08

**Authors:** Cuirong Hu, Xinqi Liao, Yi Fang, Shu Zhu, Xia Lan, Guilan Cheng

**Affiliations:** 1 Division of Internal Medicine, Institute of Integrated Traditional Chinese and Western Medicine, West China Hospital, Sichuan University/West China School of Nursing, Sichuan University, No.37, Guoxue Lane, Wuhou District, Chengdu, 610041, China, 86 18980602084

**Keywords:** chronic obstructive pulmonary disease, eHealth, home oxygen therapy, patient compliance, hospital readmission, health-related quality of life, meta-analysis, PRISMA

## Abstract

**Background:**

Chronic obstructive pulmonary disease (COPD) is a common respiratory disorder frequently requiring oxygen therapy to relieve symptoms and improve survival. In recent years, telehealth-supported interventions have emerged as promising strategies to optimize home oxygen therapy by promoting adherence, reducing hospitalizations, and enhancing health-related quality of life. However, evidence regarding their effectiveness remains inconsistent and equivocal, underscoring the need for further rigorous evaluation.

**Objective:**

This study aimed to evaluate the clinical and cost-effectiveness of telehealth-supported home oxygen therapy on adherence, hospital readmission, and health-related quality of life in patients with COPD.

**Methods:**

A comprehensive search was conducted across 6 databases (PubMed, Cochrane Central, Embase, Web of Science, PsycINFO, and CINAHL) up to October 1, 2024, and updated on April 28, 2025. Randomized controlled trials involving patients with COPD comparing telehealth-supported home oxygen therapy with usual care, and reporting outcomes on adherence, hospital readmissions, or health-related quality of life, were included. In addition, 2 reviewers independently screened the studies, extracted data, assessed the risk of bias using the Cochrane Risk of Bias 2 tool, and evaluated the certainty of evidence with the Grading of Recommendations Assessment, Development, and Evaluation approach. Meta-analyses and heterogeneity assessments were conducted using R software (R Core Team). Standardized mean differences with 95% CIs were calculated to evaluate the intervention effects under a random-effects model.

**Results:**

In total, 8 studies comprising 1275 patients were included in the review. Telehealth-supported home oxygen therapy significantly reduced hospital readmissions (standardized mean difference [SMD]=−0.40, 95% CI −0.60 to −0.21) and improved health-related quality of life (SMD=0.49, 95% CI 0.25-0.73). No significant effect was observed on therapy adherence (SMD=0.19, 95% CI −0.76 to 1.14). Furthermore, 3 economic evaluations suggested that although telehealth interventions may incur higher initial costs, they are likely to result in long-term savings by reducing hospital admissions. Sensitivity analyses confirmed the robustness of the findings for hospital readmissions and health-related quality of life, for which the quality of evidence was rated as high, whereas the evidence for therapy adherence was rated as low.

**Conclusions:**

Telehealth-supported home oxygen therapy significantly reduces hospital admissions and improves health-related quality of life in patients with COPD, but does not significantly improve therapy adherence. Tailored interventions that consider patient demographics, combined with supportive policies, may further enhance clinical outcomes. Future research should incorporate economic evaluations to better inform policy decisions regarding the implementation of telehealth-supported home oxygen therapy. However, the overall certainty of evidence is limited by study-level risk of bias, variability in intervention designs, and imprecision of effect estimates, highlighting the need for further high-quality, standardized trials.

## Introduction

Chronic obstructive pulmonary disease (COPD) is a prevalent respiratory condition characterized by persistent airflow limitation and breathing difficulties [1]. In 2020, more than 480 million people were affected globally, with an estimated prevalence of 10.6% [2]. Across all stages of the disease, patients commonly experience symptoms, complications, and treatment burdens that impair physical and psychological well-being [3]. Delayed diagnosis and late presentation are associated with poor outcomes, including reduced quality of life, frequent hospital admissions, and increased mortality [4]. COPD also imposes a significant economic burden on health care systems, with acute exacerbations accounting for the majority of related costs [5].

The most effective treatment for patients with COPD, particularly those with chronic hypercapnia or respiratory failure, remains under discussion [6]. Home oxygen therapy, including long-term oxygen therapy and home noninvasive positive pressure ventilation, is widely recognized for relieving dyspnea and improving survival [7]. However, challenges such as poor adherence, lack of timely support, and limited clinical oversight have hindered the effectiveness of home oxygen therapy [8]. Telehealth-supported interventions have gained increasing attention for improving follow-up care, enhancing clinical outcomes, and reducing health care costs, especially in the context of the COVID-19 pandemic [9,10]. Evidence indicates that, in patients with stable hypercapnic COPD, telemonitoring during home initiation of noninvasive positive pressure ventilation is noninferior to in-hospital initiation and can reduce care costs by up to 50% [11]. Telehealth-supported home oxygen therapy not only enables the collection of data on treatment compliance, ventilator parameters, and vital signs, but also functions as a digital tool for early detection and management of acute exacerbations, thereby improving clinical outcomes [12].

Owing to these advantages, the number of studies incorporating telehealth-supported interventions into routine COPD care to support symptom management has increased substantially over the past decade [13]. However, most studies in this field have focused on evaluating the acceptability, usability, and feasibility of telehealth-supported home oxygen therapy interventions for patients with COPD. These studies have primarily provided qualitative insights into patient experiences and lack robust evidence on clinical outcomes [14]. In addition, existing research has reported inconsistent findings regarding the effects of telehealth-supported programs, whether focused solely on home oxygen therapy or integrated into broader COPD management strategies [15]. For example, Jiang et al [9] reported that an internet of things–based management program improved health-related quality of life, enhanced treatment compliance, and prolonged the time to readmission due to COPD exacerbation [13]. In contrast, Baltaxe et al [16] found no significant improvements in adherence or quality of life when mobile health tools were used to support integrated care for patients receiving home noninvasive ventilation. While some focused reviews have summarized qualitative findings and patient experiences related to telehealth in home oxygen therapy for COPD [8,17], no quantitatively pooled findings from randomized controlled trials have been reported regarding its impact on therapy adherence, hospital readmissions, and health-related quality of life. Furthermore, to our knowledge, comprehensive economic evaluations assessing the cost-effectiveness of these interventions remain scarce in the current literature.

Therefore, the objectives of this meta-analysis were to (1) evaluate the clinical effectiveness of telehealth-supported home oxygen therapy interventions on adherence, hospital readmissions, and health-related quality of life in patients with COPD, based on evidence from randomized controlled trials; and (2) assess the cost-effectiveness of these interventions in this population.

## Methods

This review adhered to the guidelines set out in the PRISMA (Preferred Reporting Items for Systematic reviews and Meta-Analyses) 2020 statement (Checklist 1) and was registered in PROSPERO (CRD42024598539) [18].

### Search Strategy

We extensively searched 6 electronic databases: PubMed, Cochrane Central Register of Controlled Trials, Embase, Web of Science, PsycINFO (via EBSCO), and CINAHL (via EBSCO). This search spanned from the databases’ inception until October 1, 2024. We subsequently updated our search until April 28, 2025. For each database, we adapted Medical Subject Headings terms and database-specific keywords, including “chronic obstructive pulmonary disease,” “internet-based intervention,” “oxygen therapy,” and “randomized controlled trial.” To ensure comprehensive coverage, we manually reviewed the reference lists of previous systematic reviews and relevant publications to identify additional studies of interest. Furthermore, 2 gray literature sources were consulted to complement the database search: the World Health Organization Institutional Repository for Information Sharing and Open Grey. In addition, we screened studies in languages other than English, if available (with English titles and abstracts), to ensure no relevant literature on the topic was missed. Details of the search strategy are provided in Table S1 in Multimedia Appendix 1.

### Eligibility Criteria

Our inclusion criteria were (1) population: patients diagnosed with COPD; (2) intervention: the experimental groups received telehealth-supported home oxygen therapy, which involved internet-based platforms and tools. Home oxygen therapy included long-term oxygen treatment trials, home noninvasive ventilation, and home continuous positive airway pressure; (3) control: patients in the control groups received usual home oxygen therapy without telehealth support; (4) outcomes: studies needed to report at least one of the following indicators—adherence, hospital readmission, or health-related quality of life; and (5) study design: only randomized controlled trials published in English were included.

The exclusion criteria were (1) reviews, conference abstracts, case studies, protocols, or qualitative studies; (2) studies lacking complete data; (3) studies where full text was unavailable; and (4) duplicate publications.

### Study Selection and Data Extraction

We used EndNote version 20 (Clarivate) to remove duplicate studies, and the remaining records were screened using the web application Rayyan (Qatar Computing Research Institute) [19]. In addition, 2 authors (CH and X Liao) independently reviewed studies based on titles and abstracts, followed by a full-text review of eligible articles. Any disagreements were resolved through discussion or, if needed, consultation with a third author. Interrater agreement during the study selection process was assessed using Cohen kappa coefficient, calculated in IBM SPSS version 27.0. The level of agreement was interpreted as follows: slight (κ=0.00-0.20), fair (κ=0.21‐0.40), moderate (κ=0.41‐0.60), substantial (κ=0.61‐0.80), and almost perfect (κ=0.81‐1.00) [20].

Data extraction was also performed independently by 2 authors (CH and YF) using a standardized form. The extracted data included (1) the first author, publication year, and the country where the study was conducted; (2) participants’ age, sample size, disease stage, and baseline forced expiratory volume in 1 second; (3) the type, format, and duration of the interventions; (4) comparator interventions for the control groups; and (5) the results and outcome measures. Furthermore, data extraction focused on the effect size at the final follow-up time point reported in each study. Any discrepancies were addressed through discussion.

### Data Synthesis and Analysis

A descriptive synthesis was performed to summarize the characteristics of the included studies. Meta-analysis and heterogeneity testing were carried out using the meta package in R software (version 4.2.2; R Core Team). Given the variation in outcome measures for adherence, hospital admissions, and health-related quality of life across the included studies, the standardized mean difference (SMD) with corresponding 95% CIs was calculated to assess the intervention effects compared with control groups. Furthermore, to ensure consistent interpretation of outcomes, scores from the COPD Assessment Test were reverse-coded in accordance with the Cochrane Handbook (version 6.5) [21], so that higher scores across all studies consistently indicated better health-related quality of life.

Heterogeneity across studies was quantified using the *I*^2^ statistic and *P* value, as high heterogeneity was anticipated due to differences in specific interventions, participant characteristics, and follow-up durations [22]. Consequently, we adopted a random-effects model to obtain more conservative effect estimates. Subgroup analysis and publication bias assessments were not conducted due to the limited number of included studies [23]. Sensitivity analysis was performed using a leave-one-out approach to test the robustness of pooled results. Statistical significance was set at a *P* value <.05 in this study.

### Quality Assessment

The revised Cochrane risk of bias tool (version 2) [24] was used to evaluate the methodological quality and bias risk of the included randomized controlled trials across 5 key domains: the randomization process, deviations from intended interventions, incomplete outcome data, outcome measurement, and selective reporting of results. Each domain was assessed based on a series of signaling questions, which helped guide the assignment of bias ratings for each domain as low risk, some concerns, or high risk. The overall risk of bias for a study was determined as follows: “low risk” if all domains were rated as low risk; “some concerns” if at least 1 domain raised concerns without any being rated as high risk; and “high risk” if one or more domains were rated as high risk [24]. The assignment or intention to treat was the outcome of interest. All included studies were independently evaluated by 2 authors (CH and X Liao).

### Quality of Evidence

Two reviewers (CH and X Liao) independently assessed the quality of evidence for adherence, health-related quality of life, and readmission, rating each as high, moderate, low, or very low in accordance with the Grading of Recommendations, Assessment, Development and Evaluation (GRADE) method [25]. Furthermore, 5 categories were carefully evaluated: risk of bias, imprecision, inconsistency, indirectness, and publication bias. Given that all included studies were randomized trials, the initial quality rating was set to “high” and subsequently downgraded to moderate, low, or very low if any category was judged as “serious,” “very serious,” “likely,” or “very likely [26].” We used the web application GRADEpro Guideline Development Tool (McMaster University and Evidence Prime Inc) to create the GRADE evidence profile [27].

### Deviation From Protocol

All core review processes, including searching, screening, eligibility assessment, data extraction, and synthesis, were conducted in accordance with the registered protocol. However, the predefined outcome measures were refined during the review process. Initially, outcomes were broadly defined to include hospitalizations, exacerbations, health-related quality of life, depression, and anxiety. After study screening and data extraction, adherence, hospital readmissions, and health-related quality of life were selected as primary outcomes for meta-analysis. This refinement was based on several considerations. First, adherence, hospital readmissions, and health-related quality of life are among the most commonly reported outcomes in recent trials and reflect the growing research focus in this area. Second, these outcomes are clinically relevant in the context of telehealth-supported home oxygen therapy for COPD, as they relate to treatment compliance, disease control, and patient-centered benefits. Third, they are closely aligned with the intended functions of telemonitoring, including improving adherence, reducing preventable hospitalizations, and enhancing overall well-being.

## Results

### Search Results

**Figure 1.**
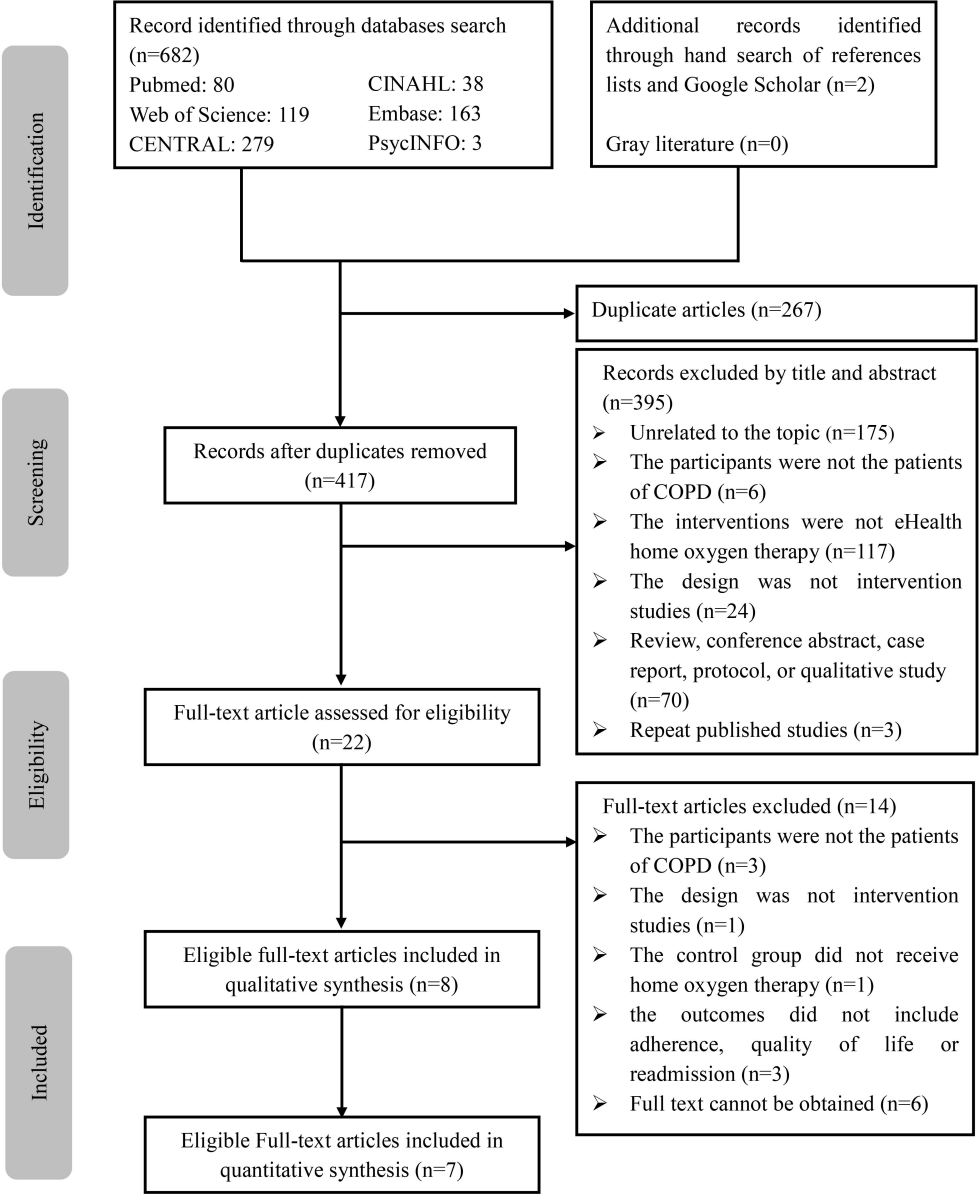
PRISMA (Preferred Reporting Items for Systematic reviews and Meta-Analyses) flow diagram. COPD: chronic obstructive pulmonary disease.

Initially, 682 records were retrieved from 6 databases, with 2 additional eligible studies identified through manual searching. After removing 267 duplicates, 417 articles remained for title and abstract screening. Next, we conducted a thorough full-text review of 22 studies, resulting in the inclusion of 8 studies in this review. The interrater agreement between the 2 reviewers (SZ and X Lan) was almost perfect, with κ scores of 0.85 (*P*<.001) for title and abstract screening and 0.94 (*P*<.001) for full-text screening. The PRISMA flowchart is shown in Figure 1.

### Characteristics of the Included Studies

#### Study Characteristics

This systematic review included 8 randomized controlled trials [9,15,28-33], conducted across 5 countries: China [9], the United States [29-31], Spain [15,28], Italy [32], and Colombia [33] with publication years ranging from 2009 to 2025 [32,33]. All included studies enrolled patients in the home setting. Further details are available in Table 1 [9,15,28-33].

**Table 1.** Characteristics of included studies.

Study	Country	Participants				Interventions				Outcomes	
		Age (y), mean (SD)	Sample size	Disease stage	Baseline FEV1^j^ (% predicted), mean (SD)	Type	Format	Duration	Control	Result	Measurement
Jiang et al [9]	China	Treatment group: 72.8 (6.5); control group: 72.7 (7.1)	Treatment group: n=73; control group: n=75	Stable	Treatment group: 25.6 (8.2); control group: 25.8 (10.0)	Home NPPV^b^	Internet of things	12 mo	Home NPPV alone	Primary: HRQoL^c^; secondary: readmission, adherence, and costs	Severe respiratory insufficiency questionnaire; medical recording data
Jódar- Sánchez et al [28]	Spain	Treatment group: 74.4 (7.6); control group: 70.8 (10.4)	Treatment group: n=24; control group: n=21	Stable	NR^d^	LTOT^e^	Application	4 mo	LTOT alone	HRQoL, hospitalizations, and costs	EuroQol-5D^f^ questionnaire; medical recording data
Leonard et al [29]	United States	Whole sample: 64.5 (5.9)	Treatment group: n=10; control group: n=10	Unstable	Whole sample: 35.2	Home NIV^g^	Call center	12 mo	Home NPPV alone	Primary: adherence; secondary: readmission	Medical recording data
Martinez et al [30]	United States	Treatment group: 64.8 (9.3); control group: 64.1 (9.8)	Treatment group: n=153; control group: n=141	NR	NR	Home CPAP^h^	Website	3 mo	Home CPAP alone	Primary: adherence; secondary: sleep quality	Medical recording data; Pittsburgh sleep quality inventory
Naranjo- Rojas et al [33]	Colombia	Treatment group: 72.1 (18.2); control group: 78.9 (13.0)	Treatment group: n=23; control group: n=22	NR	NR	Home oxygen therapy	Application	3 mo	Home oxygen therapy alone	HRQoL	Chronic Obstructive Pulmonary Disease Assessment Test
Prieto- Centurion et al [31]	United States	Proactive group: 68.0 (8.8); reactive group: 66.0 (8.4); usual care: 67.0 (9.0)	Proactive group: n=154; reactive group: n=148; usual care: n=142	NR	NR	LTOT	Telephone call	3 mo	LTOT alone	Adherence	Medical recording data
Segrelles Calvo et al [15]	Spain	Treatment group: 75.0 (9.7); control group: 72.7 (9.3)	Treatment group: n=29; control group: n=30	Stable	Treatment group: 38.3 (11.9); control group: 37.1 (10.8)	LTOT	Application	7 mo	LTOT alone	HRQoL and hospitalizations	EuroQol-5D questionnaire; medical recording data
Vitacca et al [32]	Italy	Treatment group: 61.2 (17.6); control group: 61.1 (17.4)	Treatment group: n=118; control group: n=102	NR	Treatment group: 39.0 (23.0); control group: 34.0 (16.0)	Home mechanical ventilation	Telephone call	12 mo	Home mechanical ventilation alone	Hospitalizations, exacerbations, and costs	Medical recording data

a FEV1: forced expiratory volume in 1 second.

b NPPV: noninvasive positive pressure ventilation.

c HRQoL: health-related quality of life.

d NR: not reported.

e LTOT: long-term oxygen therapy.

f EuroQol-5D questionnaire: EuroQol 5 Dimensions Questionnaire.

g NIV: noninvasive ventilation.

h CPAP: continuous positive airway pressure.

### Characteristics of Patients

A total of 1275 individuals with COPD participated in the studies, with 732 receiving telehealth-supported home oxygen therapy and 543 receiving only home oxygen therapy. The mean age of participants ranged from 61 to 79 years [32,33]. Furthermore, 3 studies focused on patients with stable COPD (no recent exacerbation) [9,15,28], while 1 study included patients with recent exacerbation [29]. The baseline forced expiratory volume in 1 second (% predicted) ranged from 25 to 39 [9,32].

### Characteristics of Telehealth-Supported Home Oxygen Therapy

The telehealth interventions were organized by delivery format, including internet of things [9], applications [15,28,33], telephone calls [31,32], call centers [29], and websites [30]. Intervention durations varied from 3 to 12 months. Regarding specific types of home oxygen therapy, 3 studies examined the effects of long-term oxygen therapy [15,28,31], while the remaining studies focused on noninvasive ventilation [9,29], continuous positive airway pressure [30], and home mechanical ventilation [32].

Generally, the telehealth-supported home oxygen interventions in the included studies aimed to provide real-time remote monitoring of each patient’s clinical information and ventilator parameters. In cases of alarms or alerts, health care professionals would contact patients by telephone or other internet-based tools, offering preliminary assessments [9,15,28,29,32], promoting adherence [9,29-31,33], enhancing symptom management [9,15,28-33], addressing problems on a case-by-case basis, and arranging emergency home visits when necessary.

### Characteristics of Controls

Participants in the control groups received home oxygen therapy without telehealth support, along with standard home oxygen management throughout the study period in all included studies [9,15,28-33].

### Outcome Measures

The outcomes assessed were adherence, readmission, and health-related quality of life. Health-related quality of life was measured using 3 scales: the Severe Respiratory Insufficiency Questionnaire [9], the COPD Assessment Test [33], and the EuroQol 5 Dimensions Questionnaire [15,28]. Data on adherence and readmission were objectively obtained from medical records.

### Meta-Analysis Results

#### Adherence

**Figure 2.**
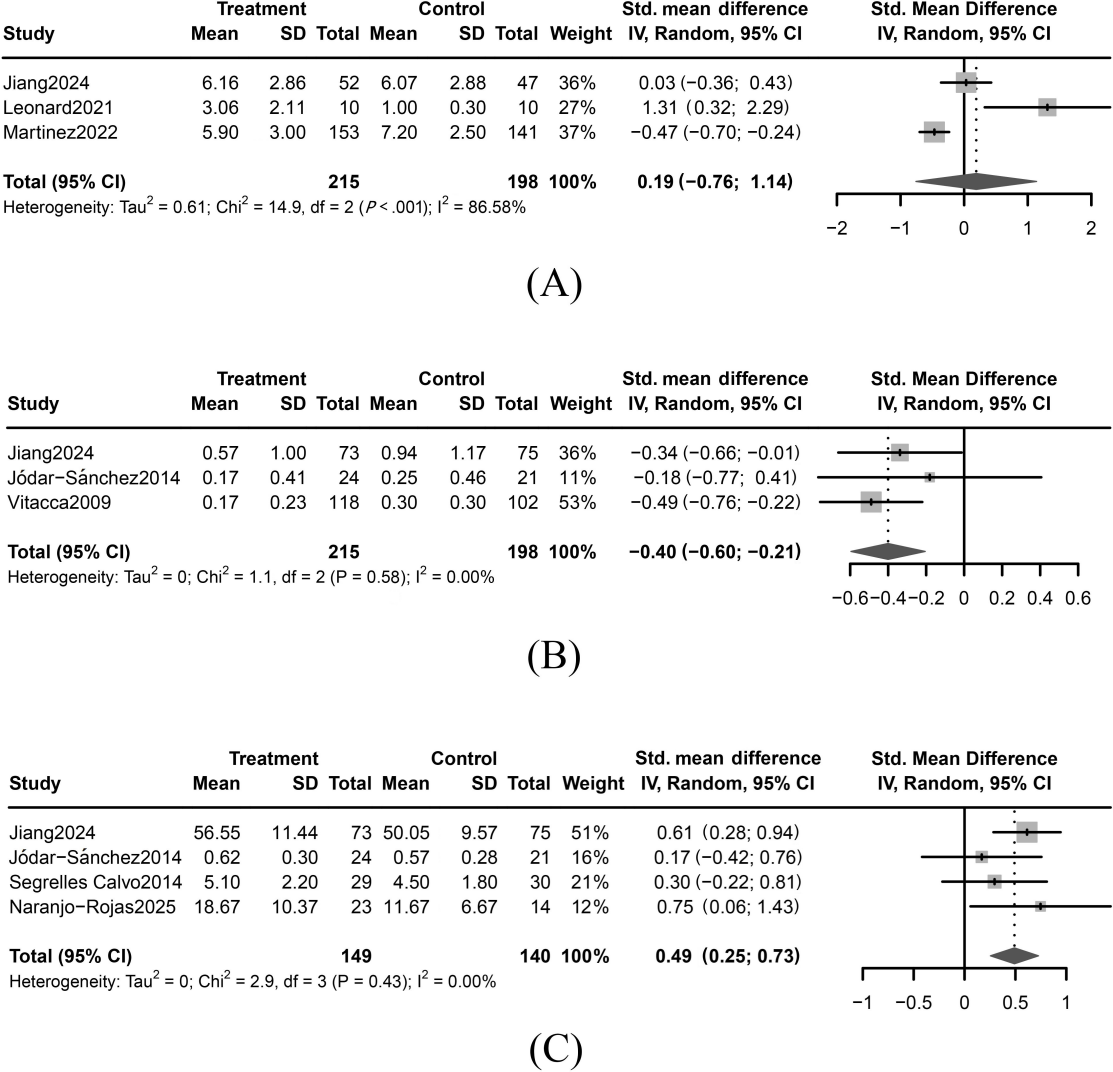
Forest plot of the effect of telehealth-supported home oxygen therapy on (**A**) adherence, (**B**) hospital readmission, and (**C**) health-related quality of life [9,15,28-30,32,33]. Std.: standardized.

In total, 3 studies investigated the effectiveness of telehealth-supported home oxygen therapy on adherence among individuals with COPD [9,29,30]. Given the high heterogeneity across studies, a random effects model was applied for analysis. The aggregated findings indicated no significant improvement in adherence after receiving telehealth-supported home oxygen therapy (standardized mean difference [SMD]=0.19, 95% CI −0.76 to 1.14) (Figure 2A [9,29,30]). Sensitivity analysis demonstrated that removing any individual study did not alter the overall trend; however, excluding 1 specific study [29] shifted the effect from positive to negative, likely due to its focus on a smaller, less stable COPD sample (Figure S1 in Multimedia Appendix 1).

#### Hospital Readmission

In total, 3 studies evaluated the effectiveness of telehealth-supported home oxygen therapy on hospital readmission among individuals with COPD [9,28,32]. A random effects model was applied due to study variability. The combined results demonstrated a statistically significant effect of telehealth-supported home oxygen therapy in reducing hospital readmission for individuals with COPD (SMD=−0.40, 95% CI −0.60 to −0.21) (Figure 2B [9,28,32]). Sensitivity analysis confirmed the stability of these results (Figure S2 in Multimedia Appendix 1).

#### Health-Related Quality of Life

In total, 4 studies examined the impact of telehealth-supported home oxygen therapy on health-related quality of life among individuals with COPD [9,15,28,33]. A random effects model was used to calculate the pooled effect size. The combined results indicated a significant improvement in health-related quality of life for individuals receiving telehealth-supported home oxygen therapy (SMD=0.49, 95%CI 0.25-0.73) (Figure 2C [9,15,28,33]). Sensitivity analysis showed that the results were stable (Figure S3 in Multimedia Appendix 1).

### Economic Outcomes

In total, 3 studies reported on the cost of telehealth-supported home oxygen therapy interventions [9,28,32]. Jiang et al [9] conducted a cost-effectiveness analysis comparing internet-of-things–based management of home noninvasive ventilation for patients with COPD to standard management. The study found a marginal increase in quality-adjusted life years for the intervention group (0.45, 95% CI 0.39‐0.52) compared with the control group (0.44, 95% CI 0.38‐0.51). Jódar-Sánchez et al [28] performed a cost-utility analysis from the health care payer perspective, which demonstrated an incremental cost-effectiveness ratio of €223,726 per quality-adjusted life year for the telehealth group compared with the control group (a currency exchange rate of €1=US $1.17 is applicable). Vitacca et al [32] also conducted a cost-effectiveness analysis, including the costs of telemedicine services, health care services, and private expenses. The study found that after accounting for the cost of tele-assistance, the average overall cost per patient was 33% lower than for usual care, indicating significant cost savings without compromising care quality. The details of the economic evaluations are presented in Table 2 [9,28,32].

**Table 2.** Economic evaluation of telehealth-supported home oxygen therapy interventions.

Study	Type of economic evaluation interventions	Costs included	Economic outcome	Finding
Jiang et al [9]	Cost-effectiveness analysis	Costs included hospitalization due to exacerbations, telemedicine equipment, telephone consultations, home visits by health care professionals, ventilator equipment, and oxygen supply.	Total cost (¥)^a^ NPPV^b^ plus IOT^c^: 11,094 NPPV alone: 14,922 Stable COPD^d^ costs (¥) NPPV plus IOT: 9524 NPPV alone: 7451 Total QALYs^e^ NPPV plus IOT: 0.45 NPPV alone: 0.44	The incremental cost- effectiveness ratio comparing the intervention group to the control group was ¥208,551 per QALY, which is below 3 times the GDP^f^ per capita.
Jódar- Sánchez et al [28]	Cost-utility analysis	Costs included specialized care, hospitalization for COPD and other chronic diseases, clinical call center services, mobile phone use, medical parameter monitoring, and tele-modem hub equipment.	Average cost (€)^g^ TG^h^ without comorbidity: 855 CG^i^ without comorbidity: 1354 Average QALYs TG without comorbidity: 0.0288 CG without comorbidity: 0.0082	The analysis yielded an incremental cost-effectiveness ratio of €223,726 per QALY
Vitacca et al [32]	Cost-effectiveness analysis	Costs included telemedicine services (call center, pulse oximetry device), health care services (hospitalizations, admissions, and medical visits), and private expenses (transportation).	Total cost (€) TA^j^: 8907 Control: 14,728	After deduction of TA costs, the average overall cost for each patient was 33% less than that for usual care.

a A currency exchange rate of ¥1=US $0.13961 is applicable.

b NPPV, non-invasive positive pressure ventilation.

c IOT: internet of things.

d COPD: chronic obstructive pulmonary disease.

e QALYs: quality-adjusted life years.

f GDP: gross domestic product.

g A currency exchange rate of €1=US $1.17600 is applicable.

h TG: telehealth group.

i CG: control group.

j TA: tele-assistance.

### Risk of Bias and Quality of Evidence

**Figure 3.**
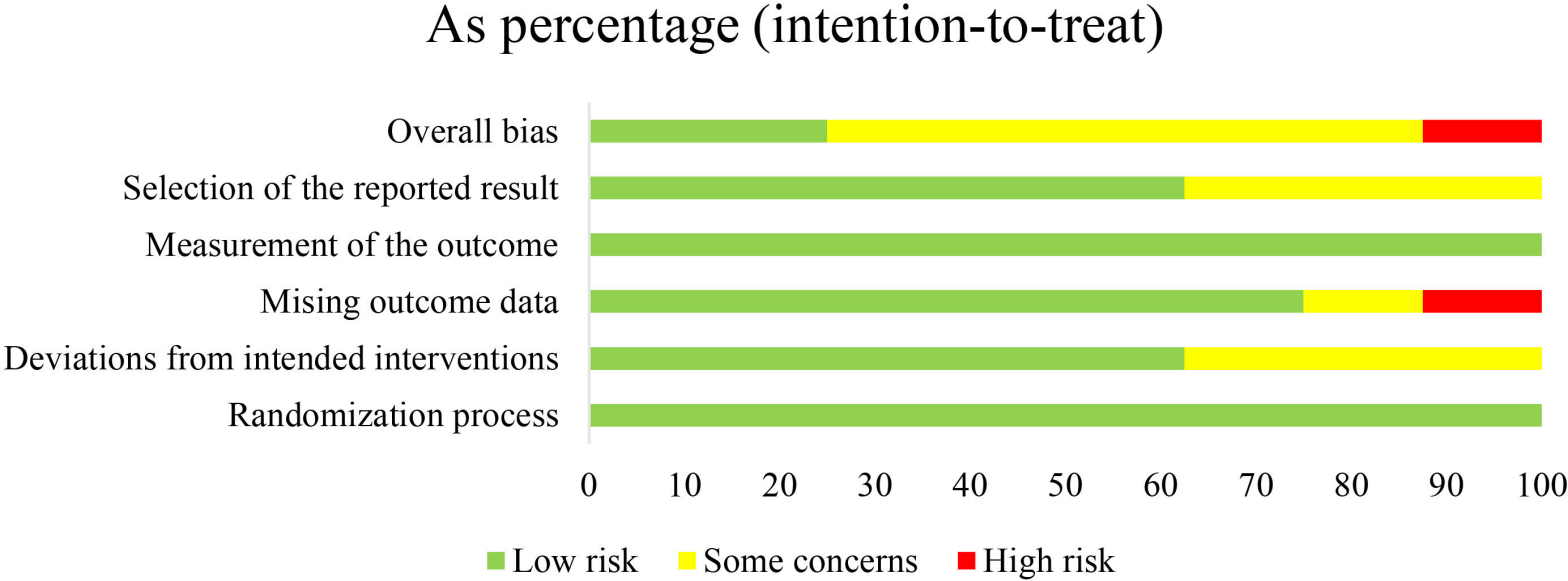
Risk of bias summary of the included studies.

According to the revised Cochrane risk-of-bias tool, 2 studies was judged to have a low risk of bias [9,33]. Furthermore, 5 studies were rated as having “some concerns” due to incomplete reporting of intervention allocation details, raising concerns about deviations from intended interventions [15,28,32]. In addition, missing outcome data and selective reporting were identified as sources of bias [29,30]. In addition, 1 study was appraised as high risk for missing adherence data due to incomplete oxygen equipment worksheets and follow-up records [31]. The summaries of risk of bias are provided in Figure 3 and Figure S4 in Multimedia Appendix 1.

According to the GRADE approach, the quality of evidence was rated as low for adherence, high for hospital readmission, and high for health-related quality of life. The downgrading factors primarily stemmed from the risk of bias in included studies, imprecision of results, and heterogeneity across studies. The evaluation details are presented in Table S2 in Multimedia Appendix 1 .

## Discussion

### Summary of Main Results

To our knowledge, this study is the first systematic review and meta-analysis of randomized controlled trials evaluating the clinical and cost-effectiveness of telehealth-supported home oxygen therapy in terms of adherence, hospital readmissions, and health-related quality of life. This systematic review of randomized controlled trials included a total of 1275 patients with COPD. Compared with control groups, telehealth-supported home oxygen therapy interventions reduced hospital readmissions and improved health-related quality of life but did not significantly increase adherence. Given that the overall methodological quality of most included studies raised some concerns or indicated a high risk of bias, and that the quality of evidence for adherence was low, more high-quality randomized controlled trials are needed to draw robust conclusions.

### Principal Findings

#### Adherence

The results of this meta-analysis showed that telehealth-supported home oxygen therapy interventions did not significantly improve adherence among patients with COPD, which is consistent with previous studies [9,16]. Adherence to home oxygen therapy is a critical factor influencing quality of life, dyspnea, exercise capacity, and mortality in patients with COPD [34]. However, all 3 included studies reported only marginal improvements observed in the intervention groups [9,29,30]. The lack of an overall significant effect may be attributed to substantial heterogeneity among the included studies, potentially driven by differences in regional health care systems, intervention formats, and follow-up durations. While telehealth interventions offer timely access to information and support, their effectiveness may be limited in rural populations due to cultural beliefs surrounding oxygen therapy and inadequate internet infrastructure, which can impair the delivery and monitoring of home-based care [29]. In addition, the nonsignificant result may be partly influenced by 1 study that included patients with both COPD and obstructive sleep apnea [30], a population potentially less responsive to continuous positive airway pressure-based interventions. A high baseline adherence rate in that study may have also resulted in a ceiling effect. Furthermore, a 3-month intervention duration might have been insufficient to produce measurable improvements in adherence. In addition to study-level differences, several patient-related factors may also help explain the lack of improvement in adherence. Some patients may perceive early symptom relief as a reason to discontinue therapy [14], especially when ongoing behavioral or psychological support is lacking [35]. Many individuals with COPD are older adults who may face difficulties using telehealth technologies, managing oxygen equipment, or accessing eHealth resources [36]. Therefore, to enhance adherence outcomes, future telehealth-supported home oxygen interventions should be designed with greater sensitivity to patient characteristics, behavioral support needs, technological accessibility, and health care system contexts.

In addition, according to the GRADE assessment, the certainty of evidence for adherence was rated as low, primarily due to 2 downgrading domains: imprecision of results and inconsistency across studies. These limitations may have contributed to the statistically nonsignificant effect and substantial heterogeneity observed. Given the low confidence in the effect estimates, conclusions regarding adherence should be interpreted with caution.

#### Hospital Readmission

The results of this meta-analysis demonstrated that telehealth-supported home oxygen therapy interventions significantly reduced hospital readmissions among patients with COPD, consistent with findings from previous studies [13]. These interventions enable health care professionals to telemonitor clinical information and ventilator parameters, which are more accurate and timely than those in the control group [9,37]. In addition, health management teams conducted telephone check-ins and home visits in response to alerts or emergencies, ensuring early detection of exacerbations in patients with COPD [38]. Even in cases where no direct responses are made through telehealth interventions, patient-generated health data allow health care teams to make informed medical decisions, thus improving care support. Furthermore, telehealth-supported home oxygen therapy interventions, facilitated by remote devices, keep patients engaged, meet their needs, and provide access to assistance anytime and anywhere. This is particularly valuable for patients with mobility limitations or those living in rural areas, where regular hospital visits may be challenging [39]. Systematic monitoring, early identification, and prompt management of acute needs contribute to reducing hospital readmissions among patients with COPD [40,41]. However, the limited number of studies and potentially small study effects may have influenced the findings. Future research should prioritize large-scale randomized controlled trials to provide more robust evidence on the impact of telehealth-supported home oxygen therapy. In addition, exploring potential moderators, such as disease severity and patient demographics, will help clarify how these factors influence the intervention’s effectiveness in reducing hospital readmissions.

#### Health-Related Quality of Life

The meta-analysis found that telehealth-supported home oxygen therapy significantly improved health-related quality of life, which is in agreement with previous studies [9]. Improvements were observed across several domains, including respiratory complaints, pulmonary rehabilitation, physical functioning, and psychological well-being [9,28]. These interventions help meet the needs of patients with COPD by enhancing their ability to manage physical and psychological symptoms while providing timely access to health care providers, thus improving their overall health-related quality of life [42]. Beyond symptom management, patients also gain valuable knowledge on coping with COPD, which enhances their confidence, satisfaction, and quality of life [42]. Furthermore, the integration of patient-generated data into electronic health records allows for more efficient tracking and monitoring, supporting timely clinical actions and improving patient satisfaction with their care [43]. For low-income populations, where access to regular health care may be limited due to financial and logistical barriers, telehealth interventions can offer a cost-effective solution that improves health outcomes [44]. However, while these findings are promising, more high-quality clinical trials are needed to assess the long-term impact of telehealth-supported home oxygen therapy on health-related quality of life. Future studies should explore the mechanisms underlying these improvements and examine specific domains of health-related quality of life to refine interventions for patients with COPD.

### Economic Findings

This review analyzed the cost-effectiveness of telehealth-supported home oxygen therapy for patients with COPD. Furthermore, 3 studies highlighted the significant economic burden that COPD imposes on health care systems [9,28,32]. Jiang et al [9] found that the average total 1-year cost per patient for stable COPD in the intervention group was ¥9524, compared with ¥7451 in the control group. However, the overall costs in the intervention group were lower, with the cost difference primarily attributed to the expenses related to health care professional contacts, telemedicine tools, and the effectiveness of internet-of-things–based management of home noninvasive ventilation. Jódar-Sánchez et al [28] adopted a health care payer perspective, meaning that cost savings would be fully absorbed by the health care system. However, this perspective overlooks out-of-pocket costs borne by patients with COPD and their families, such as caregiving expenses [45]. Therefore, future intervention studies need to conduct a more comprehensive cost analysis. Vitacca et al [32] concluded that in patients with chronic respiratory failure who are on oxygen or home mechanical ventilation, nurse-centered tele-assistance prevents hospitalizations and is cost-effective. This study considered costs from a broader societal perspective, including those borne by the health care system, patients, and other relevant parties.

Overall, all studies demonstrated that although the costs of maintaining telemedicine equipment, telephone consultations, and home visits by health care professionals were higher compared with the control group, reduced hospitalizations due to exacerbations led to lower overall health care costs [9,28,32]. This suggests that, despite higher initial costs, telehealth and home oxygen therapy interventions may result in long-term cost savings by preventing hospital admissions.

### Limitations

This study has several limitations. First, heterogeneity may have been introduced due to variations in study characteristics, including intervention formats, follow-up lengths, COPD severity, and outcome measures. Second, the economic analysis was limited by inconsistent cost perspectives and the lack of consideration for indirect costs such as caregiver burden and patient out-of-pocket expenses. Future research should adopt standardized frameworks that incorporate both direct and indirect costs to more accurately evaluate the financial impact of telehealth-supported home oxygen therapy. Third, the methodological quality of the included studies varied, with several studies showing some concerns or high risk of bias due to incomplete data and selective reporting. The certainty of evidence for adherence was low, which may reduce the reliability of this outcome. Future studies should employ rigorous randomized controlled trial designs, including blinding, complete outcome reporting, and standardized adherence measurement, to strengthen the quality of evidence. Finally, although this review focused on key clinical and economic outcomes, patient-centered factors such as satisfaction and psychological burden were underexplored. The lack of patient experience data limits understanding of adherence barriers. Future studies should consider qualitative or mixed methods approaches to better reflect patient perspectives and enhance real-world applicability.

### Conclusions

Our review revealed that telehealth-supported home oxygen therapy significantly reduced hospital admissions and improved health-related quality of life, but had no significant effect on improving adherence among people living with COPD. High-quality longitudinal studies are needed to further examine whether this intervention can enhance adherence in patients with COPD. In addition to introducing relevant policies and regulations at higher levels, tailored telehealth-supported home oxygen therapy, based on demographic characteristics of patients with COPD, is warranted to improve their well-being. Furthermore, including economic analyses in future research would help policy makers make informed decisions regarding the implementation of telehealth-supported home oxygen therapy.

## Supplementary material

10.2196/73010 Multimedia Appendix 1 Supplementary tables and figures.

10.2196/73010 Checklist 1 PRISMA (Preferred Reporting Items for Systematic reviews and Meta-Analyses) checklist.

## Abbreviations

COPD: chronic obstructive pulmonary disease
GRADE: Grading of Recommendations, Assessment, Development and Evaluation
PRISMA: Preferred Reporting Items for Systematic reviews and Meta-Analyses
SMD: standardized mean difference
