# Supplementary material for: Clinical and Cost-Effectiveness of Telehealth-Supported Home Oxygen Therapy on Adherence, Hospital Readmission, and Health-Related Quality of Life in Patients With Chronic Obstructive Pulmonary Disease: Systematic Review and Meta-Analysis of Randomized Controlled Trials
Source: J Med Internet Res. 2025 Jul 8;27:e73010. doi: 10.2196/73010 (PMC12262104; doi:10.2196/73010)
Supplement: Multimedia Appendix 1 [file jmir-v27-e73010-s001.docx]

**Contents of supplementary appendix**

| Table S1 | Search strategy | Page 2 |
| --- | --- | --- |
| Figure S1 | Sensitivity analysis for adherence | Page 9 |
| Figure S2 | Sensitivity analysis for hospital readmission | Page 10 |
| Figure S3 | Sensitivity analysis for health-related quality of life | Page 11 |
| Figure S4 | Risk of bias of the included studies | Page 12 |
| Table S2 | GRADE summary of the quality of the evidence for adherence, health-related quality of life and hospital readmission | Page 13 |

**Table S1.** Search strategy.

| **#** | **PubMed** |
| --- | --- |
| 1 | "pulmonary disease, chronic obstructive"[MeSH Terms] |
| 2 | COPD[Title/Abstract] OR COAD[Title/Abstract] OR "chronic airflow obstruction*"[Title/Abstract] OR "chronic airway obstruction*"[Title/Abstract] OR "chronic obstructive airway*"[Title/Abstract] OR "chronic obstructive bronch*"[Title/Abstract] OR "chronic bronch*"[Title/Abstract] OR "chronic obstructive lung*"[Title/Abstract] OR "chronic obstructive pulmonar*"[Title/Abstract] OR "chronic obstructive respira*"[Title/Abstract] OR "obstructive lung diseas*"[Title/Abstract] OR "obstructive respiratory tract*"[Title/Abstract] OR "bronchitis chronic*"[Title/Abstract] OR chronic pulmonary obstructi*[Title/Abstract] OR "pulmonary emphysem*"[Title/Abstract] OR "focal emphysem*"[Title/Abstract] OR "panacinar emphysem*"[Title/Abstract] OR "panlobular emphysema*"[Title/Abstract] OR "centriacinar emphysem*"[Title/Abstract] OR "centrilobular emphysema*"[Title/Abstract] OR "bullous emphysem*"[Title/Abstract] OR "intrapulmonary interstitial emphysem*"[Title/Abstract] OR "lobular emphysem*"[Title/Abstract] OR "lung interstitial emphysem*"[Title/Abstract] OR pneumonectasia*[Title/Abstract] |
| **3** | **#1 OR #2** |
| 4 | "Internet-Based Intervention"[MeSH Terms] |
| 5 | "Telemedicine"[MeSH Terms] |
| 6 | "Telerehabilitation"[MeSH Terms] |
| 7 | "internet*"[Title/Abstract] OR "web based*"[Title/Abstract] OR "online*"[Title/Abstract] OR "mobile*"[Title/Abstract] OR "remote*"[Title/Abstract] OR "digital*"[Title/Abstract] OR "virtual medicine*"[Title/Abstract] OR "mhealth*"[Title/Abstract] OR "ehealth*"[Title/Abstract] OR "telehealth*"[Title/Abstract] OR "telemedicine*"[Title/Abstract] OR "telecare*"[Title/Abstract] OR "telerehabilitation*"[Title/Abstract] OR "telemonitor*"[Title/Abstract] OR "telecommunication*"[Title/Abstract] OR "telemanagement*"[Title/Abstract] OR "virtual rehabilitation*"[Title/Abstract] OR "tele rehabilitation*"[Title/Abstract] OR "tele care*"[Title/Abstract] OR "phone*"[Title/Abstract] |
| **8** | **#4 OR #5 OR #6 OR #7** |
| 9 | "Oxygen Inhalation Therapy"[MeSH Terms] |
| 10 | "oxygen*"[Title/Abstract] OR "O_2_"[Title/Abstract] OR "noninvasive ventilation*"[Title/Abstract] OR "mechanical ventilation*"[Title/Abstract] OR "high flow nasal cannula*"[Title/Abstract] |
| **11** | **#9 OR #10** |
| 12 | "Randomized Controlled Trials as Topic"[MeSH Terms] |
| 13 | "Randomized Controlled Trial"[Publication Type] OR "randomized controlled trial*"[Title/Abstract] OR "RCT"[Title/Abstract] OR "random*"[Title/Abstract] OR "allocate*"[Title/Abstract] OR "assign*"[Title/Abstract] |
| **14** | **#12 OR #13** |
| **15** | **#3 AND #8 AND #11 AND #14** |
|  |  |
| **#** | **Cochrane Central Register of Controlled Trials** |
| 1 | MeSH descriptor: [Pulmonary Disease, Chronic Obstructive] explode all trees |
| 2 | (COPD OR COAD OR chronic airflow obstruction* OR chronic airway obstruction* OR chronic obstructive airway* OR chronic obstructive bronch* OR chronic bronch* OR chronic obstructive lung* OR chronic obstructive pulmonar* OR chronic obstructive respira* OR obstructive lung diseas* OR obstructive respiratory tract* OR bronchitis chronic* OR chronic pulmonary obstructi* OR pulmonary emphysem* OR focal emphysem* OR panacinar emphysem* OR panlobular emphysema* OR centriacinar emphysem* OR centrilobular emphysema* OR bullous emphysem* OR intrapulmonary interstitial emphysem* OR lobular emphysem* OR lung interstitial emphysem* OR pneumonectasia*):ti,ab,kw |
| **3** | **#1 OR #2** |
| 4 | MeSH descriptor: [Internet-Based Intervention] explode all trees |
| 5 | MeSH descriptor: [Telerehabilitation] explode all trees |
| 6 | (internet* OR web based* OR online* OR mobile* OR remote* OR digital* OR virtual medicine* OR mhealth* OR ehealth* OR telehealth* OR telemedicine* OR telecare* OR telerehabilitation* OR telemonitor* OR telecommunication* OR telemanagement* OR virtual rehabilitation* OR tele rehabilitation* OR tele care* OR phone*):ti,ab,kw |
| **7** | **#4 OR #5 OR #6** |
| 8 | MeSH descriptor: [Oxygen Inhalation Therapy] explode all trees |
| 9 | (oxygen* OR O_2_ OR noninvasive ventilation* OR mechanical ventilation* OR high flow nasal cannula*):ti,ab,kw |
| **10** | **#8 OR #9** |
| 11 | MeSH descriptor: [Randomized Controlled Trials as Topic] explode all trees |
| 12 | (randomized controlled trial* OR RCT OR random* OR allocate* OR assign*):ti,ab,kw |
| **13** | **#11 OR #12** |
| **14** | **#3 AND #7 AND #10 AND #13** |
|  |  |
| **#** | **Embase** |
| 1 | 'chronic obstructive lung disease'/exp |
| 2 | copd:ti,ab,kw OR coad:ti,ab,kw OR 'chronic airflow obstruction*':ti,ab,kw OR 'chronic airway obstruction*':ti,ab,kw OR 'chronic obstructive airway*':ti,ab,kw OR 'chronic obstructive bronch*':ti,ab,kw OR 'chronic bronch*':ti,ab,kw OR 'chronic obstructive lung*':ti,ab,kw OR 'chronic obstructive pulmonar*':ti,ab,kw OR 'chronic obstructive respira*':ti,ab,kw OR 'obstructive lung diseas*':ti,ab,kw OR 'obstructive respiratory tract*':ti,ab,kw OR 'bronchitis chronic*':ti,ab,kw OR 'chronic pulmonary obstructi*':ti,ab,kw OR 'pulmonary emphysem*':ti,ab,kw OR 'focal emphysem*':ti,ab,kw OR 'panacinar emphysem*':ti,ab,kw OR 'panlobular emphysema*':ti,ab,kw OR 'centriacinar emphysem*':ti,ab,kw OR 'centrilobular emphysema*':ti,ab,kw OR 'bullous emphysem*':ti,ab,kw OR 'intrapulmonary interstitial emphysem*':ti,ab,kw OR 'lobular emphysem*':ti,ab,kw OR 'lung interstitial emphysem*':ti,ab,kw OR pneumonectasia*:ti,ab,kw |
| **3** | **#1 OR #2** |
| 4 | 'web-based intervention'/exp |
| 5 | 'telemedicine'/exp |
| 6 | internet*:ti,ab,kw OR 'web based*':ti,ab,kw OR online*:ti,ab,kw OR mobile*:ti,ab,kw OR remote*:ti,ab,kw OR digital*:ti,ab,kw OR 'virtual medicine*':ti,ab,kw OR mhealth*:ti,ab,kw OR ehealth*:ti,ab,kw OR telehealth*:ti,ab,kw OR telemedicine*:ti,ab,kw OR telecare*:ti,ab,kw OR telerehabilitation*:ti,ab,kw OR telemonitor*:ti,ab,kw OR telecommunication*:ti,ab,kw OR telemanagement*:ti,ab,kw OR 'virtual rehabilitation*':ti,ab,kw OR 'tele rehabilitation*':ti,ab,kw OR 'tele care*':ti,ab,kw OR phone*:ti,ab,kw |
| **7** | **#4 OR #5 OR #6** |
| 8 | 'oxygen therapy'/exp |
| 9 | oxygen*:ti,ab,kw OR o_2_:ti,ab,kw OR 'noninvasive ventilation*':ti,ab,kw OR 'mechanical ventilation*':ti,ab,kw OR 'high flow nasal cannula*':ti,ab,kw |
| **10** | **#8 OR #9** |
| 11 | 'randomized controlled trial (topic)'/exp |
| 12 | 'randomized controlled trial*':ti,ab,kw OR rct:ti,ab,kw OR random*:ti,ab,kw OR allocate*:ti,ab,kw OR assign*:ti,ab,kw |
| **13** | **#11 OR #12** |
| **18** | **#3 AND #7 AND #10 AND #13** |
|  |  |
| **#** | **Web of science** |
| 1 | TS = (COPD OR COAD OR chronic airflow obstruction* OR chronic airway obstruction* OR chronic obstructive airway* OR chronic obstructive bronch* OR chronic bronch* OR chronic obstructive lung* OR chronic obstructive pulmonar* OR chronic obstructive respira* OR obstructive lung diseas* OR obstructive respiratory tract* OR bronchitis chronic* OR chronic pulmonary obstructi* OR pulmonary emphysem* OR focal emphysem* OR panacinar emphysem* OR panlobular emphysema* OR centriacinar emphysem* OR centrilobular emphysema* OR bullous emphysem* OR intrapulmonary interstitial emphysem* OR lobular emphysem* OR lung interstitial emphysem* OR pneumonectasia*) |
| 2 | TS = (internet* OR web based* OR online* OR mobile* OR remote* OR digital* OR virtual medicine* OR mhealth* OR ehealth* OR telehealth* OR telemedicine* OR telecare* OR telerehabilitation* OR telemonitor* OR telecommunication* OR telemanagement* OR virtual rehabilitation* OR tele rehabilitation* OR tele care* OR phone*) |
| 3 | TS = (oxygen* OR O_2_ OR noninvasive ventilation* OR mechanical ventilation* OR high flow nasal cannula*) |
| **4** | **#1 AND #2 AND #3** |
| 5 | TS = (randomized controlled trial* OR RCT OR random* OR allocate* OR assign*) |
| **6** | **#4 AND #5** |
|  |  |
| **S** | **CINAHL via EBSCO** |
| 1 | MH Pulmonary Disease, Chronic Obstructive |
| 2 | TI ( COPD OR COAD OR chronic airflow obstruction* OR chronic airway obstruction* OR chronic obstructive airway* OR chronic obstructive bronch* OR chronic bronch* OR chronic obstructive lung* OR chronic obstructive pulmonar* OR chronic obstructive respira* OR obstructive lung diseas* OR obstructive respiratory tract* OR bronchitis chronic* OR chronic pulmonary obstructi* OR pulmonary emphysem* OR focal emphysem* OR panacinar emphysem* OR panlobular emphysema* OR centriacinar emphysem* OR centrilobular emphysema* OR bullous emphysem* OR intrapulmonary interstitial emphysem* OR lobular emphysem* OR lung interstitial emphysem* OR pneumonectasia* ) OR AB ( COPD OR COAD OR chronic airflow obstruction* OR chronic airway obstruction* OR chronic obstructive airway* OR chronic obstructive bronch* OR chronic bronch* OR chronic obstructive lung* OR chronic obstructive pulmonar* OR chronic obstructive respira* OR obstructive lung diseas* OR obstructive respiratory tract* OR bronchitis chronic* OR chronic pulmonary obstructi* OR pulmonary emphysem* OR focal emphysem* OR panacinar emphysem* OR panlobular emphysema* OR centriacinar emphysem* OR centrilobular emphysema* OR bullous emphysem* OR intrapulmonary interstitial emphysem* OR lobular emphysem* OR lung interstitial emphysem* OR pneumonectasia* ) |
| **3** | **S1 OR S2** |
| 4 | MH Internet-Based Intervention |
| 5 | MH Telemedicine |
| 6 | MH Telerehabilitation |
| 7 | MH Telehealth |
| 8 | TI ( internet* OR web based* OR online* OR mobile* OR remote* OR digital* OR virtual medicine* OR mhealth* OR ehealth* OR telehealth* OR telemedicine* OR telecare* OR telerehabilitation* OR telemonitor* OR telecommunication* OR telemanagement* OR virtual rehabilitation* OR tele rehabilitation* OR tele care* OR phone* ) OR AB ( internet* OR web based* OR online* OR mobile* OR remote* OR digital* OR virtual medicine* OR mhealth* OR ehealth* OR telehealth* OR telemedicine* OR telecare* OR telerehabilitation* OR telemonitor* OR telecommunication* OR telemanagement* OR virtual rehabilitation* OR tele rehabilitation* OR tele care* OR phone* ) |
| **9** | **S4 OR S5 OR S6 OR S7 OR S8** |
| 10 | MH Oxygen Therapy |
| 11 | TI (oxygen* OR O_2_ OR noninvasive ventilation* OR mechanical ventilation* OR high flow nasal cannula*) OR AB (oxygen* OR O_2_ OR noninvasive ventilation* OR mechanical ventilation* OR high flow nasal cannula* ) |
| **12** | **S10 OR S11** |
| 13 | MH Randomized Controlled Trials |
| 14 | TI ( randomized controlled trial* OR RCT OR random* OR allocate* OR assign* ) OR AB ( randomized controlled trial* OR RCT OR random* OR allocate* OR assign* ) |
| **15** | **S13 OR S14** |
| **16** | **S3 AND S9 AND S12 AND S15** |
|  |  |
| **S** | **PsycINFO via EBSCO** |
| 1 | MA Chronic Obstructive Pulmonary Disease |
| 2 | TI ( COPD OR COAD OR chronic airflow obstruction* OR chronic airway obstruction* OR chronic obstructive airway* OR chronic obstructive bronch* OR chronic bronch* OR chronic obstructive lung* OR chronic obstructive pulmonar* OR chronic obstructive respira* OR obstructive lung diseas* OR obstructive respiratory tract* OR bronchitis chronic* OR chronic pulmonary obstructi* OR pulmonary emphysem* OR focal emphysem* OR panacinar emphysem* OR panlobular emphysema* OR centriacinar emphysem* OR centrilobular emphysema* OR bullous emphysem* OR intrapulmonary interstitial emphysem* OR lobular emphysem* OR lung interstitial emphysem* OR pneumonectasia* ) OR AB ( COPD OR COAD OR chronic airflow obstruction* OR chronic airway obstruction* OR chronic obstructive airway* OR chronic obstructive bronch* OR chronic bronch* OR chronic obstructive lung* OR chronic obstructive pulmonar* OR chronic obstructive respira* OR obstructive lung diseas* OR obstructive respiratory tract* OR bronchitis chronic* OR chronic pulmonary obstructi* OR pulmonary emphysem* OR focal emphysem* OR panacinar emphysem* OR panlobular emphysema* OR centriacinar emphysem* OR centrilobular emphysema* OR bullous emphysem* OR intrapulmonary interstitial emphysem* OR lobular emphysem* OR lung interstitial emphysem* OR pneumonectasia* ) |
| **3** | **S1 OR S2** |
| 4 | MA Telemedicine |
| 5 | MA Telerehabilitation |
| 6 | TI ( internet* OR web based* OR online* OR mobile* OR remote* OR digital* OR virtual medicine* OR mhealth* OR ehealth* OR telehealth* OR telemedicine* OR telecare* OR telerehabilitation* OR telemonitor* OR telecommunication* OR telemanagement* OR virtual rehabilitation* OR tele rehabilitation* OR tele care* OR phone* ) OR AB ( internet* OR web based* OR online* OR mobile* OR remote* OR digital* OR virtual medicine* OR mhealth* OR ehealth* OR telehealth* OR telemedicine* OR telecare* OR telerehabilitation* OR telemonitor* OR telecommunication* OR telemanagement* OR virtual rehabilitation* OR tele rehabilitation* OR tele care* OR phone* ) |
| **7** | **S4 OR S5 OR S6** |
| 8 | MA Oxygen Therapy |
| 9 | TI (oxygen* OR O_2_ OR noninvasive ventilation* OR mechanical ventilation* OR high flow nasal cannula* ) OR AB (oxygen* OR O_2_ OR noninvasive ventilation* OR mechanical ventilation* OR high flow nasal cannula* ) |
| **10** | **#8 OR #9** |
| 11 | MA Randomized Controlled Trials |
| 12 | TI ( randomized controlled trial* OR RCT OR random* OR allocate* OR assign* ) OR AB ( randomized controlled trial* OR RCT OR random* OR allocate* OR assign* ) |
| **13** | **S11 OR S12** |
| **14** | **S3 AND S7 AND S10 AND S13** |


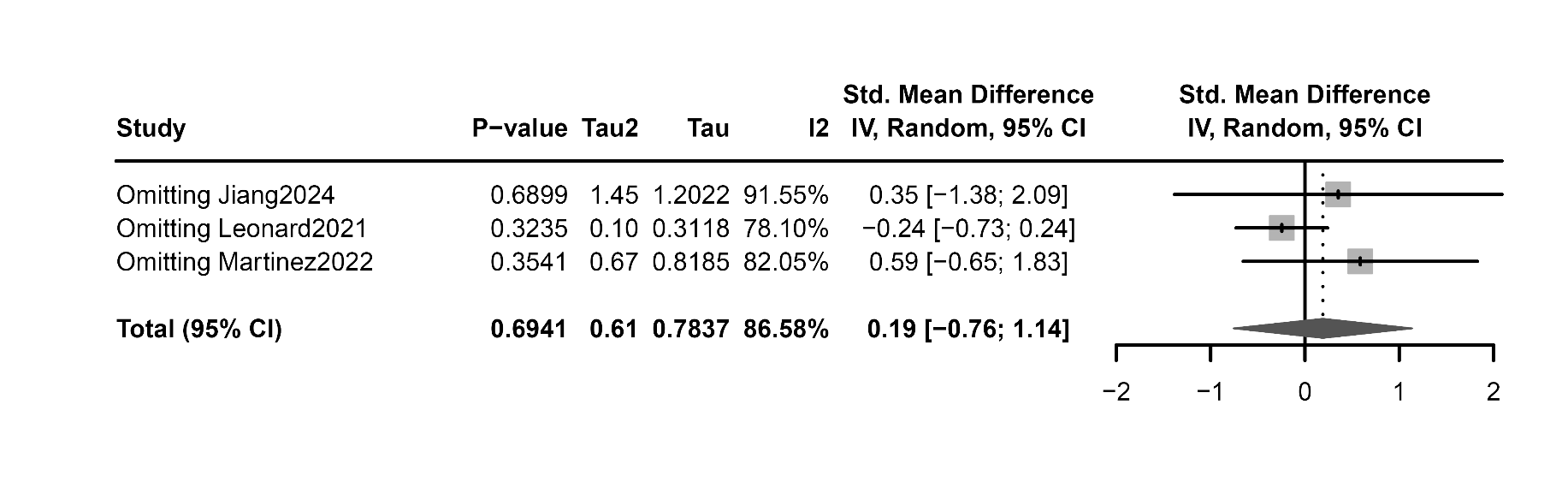
**Figure S1.** Sensitivity analysis for adherence.


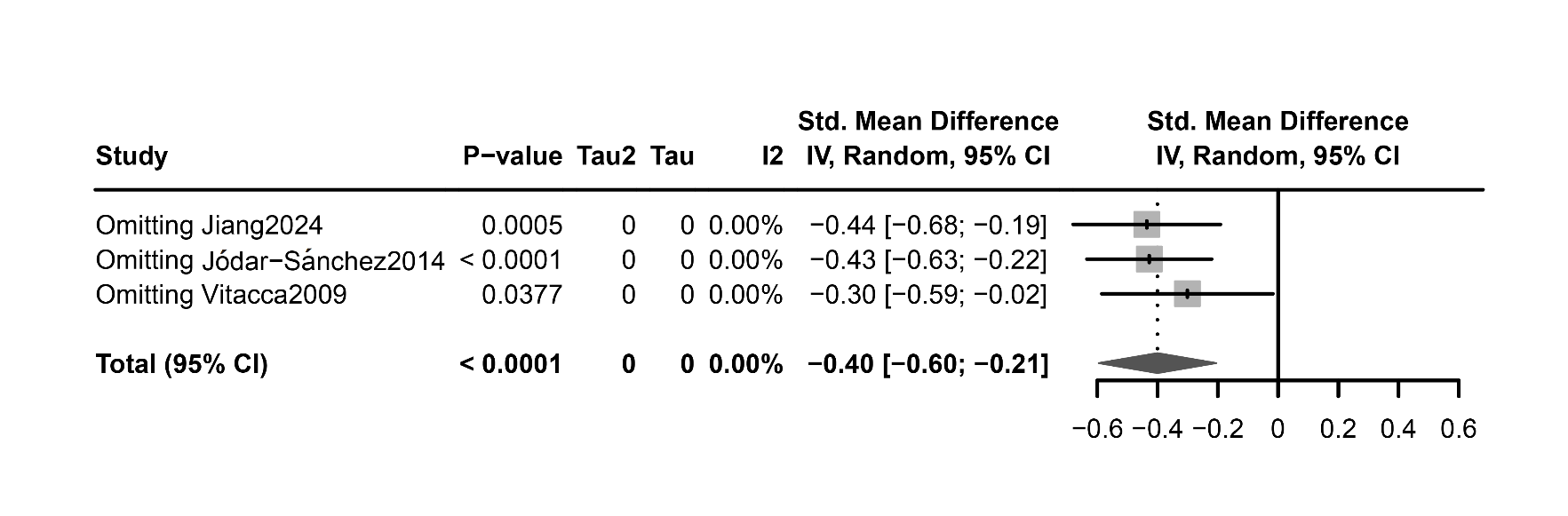


**Figure S2.** Sensitivity analysis for hospital readmission.


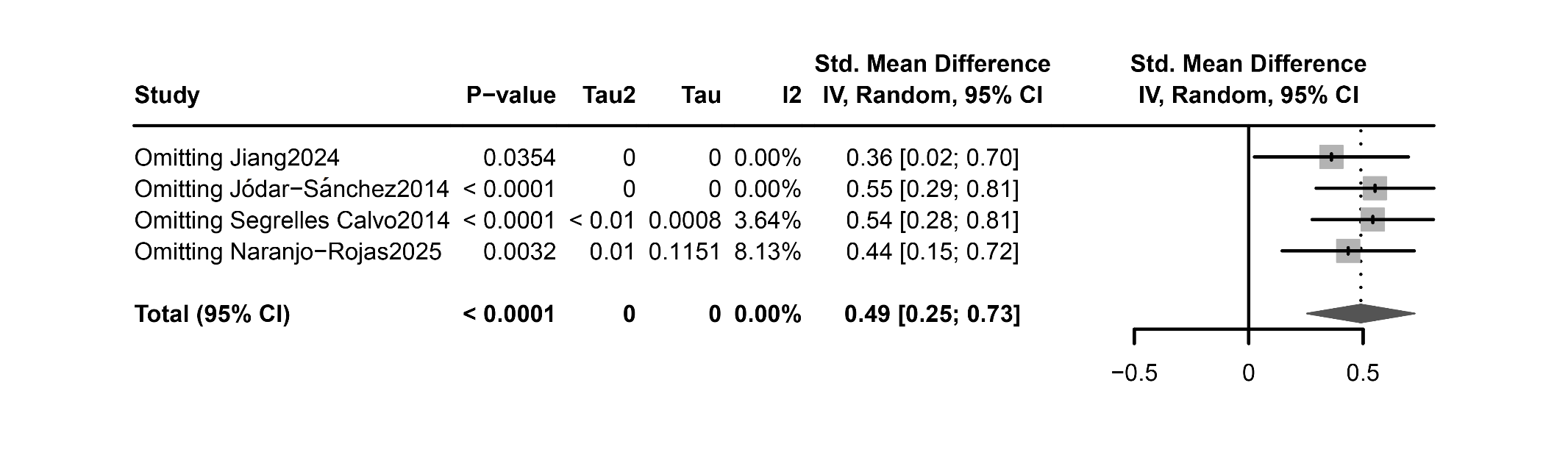
 **Figure S3.** Sensitivity analysis for health-related quality of life.


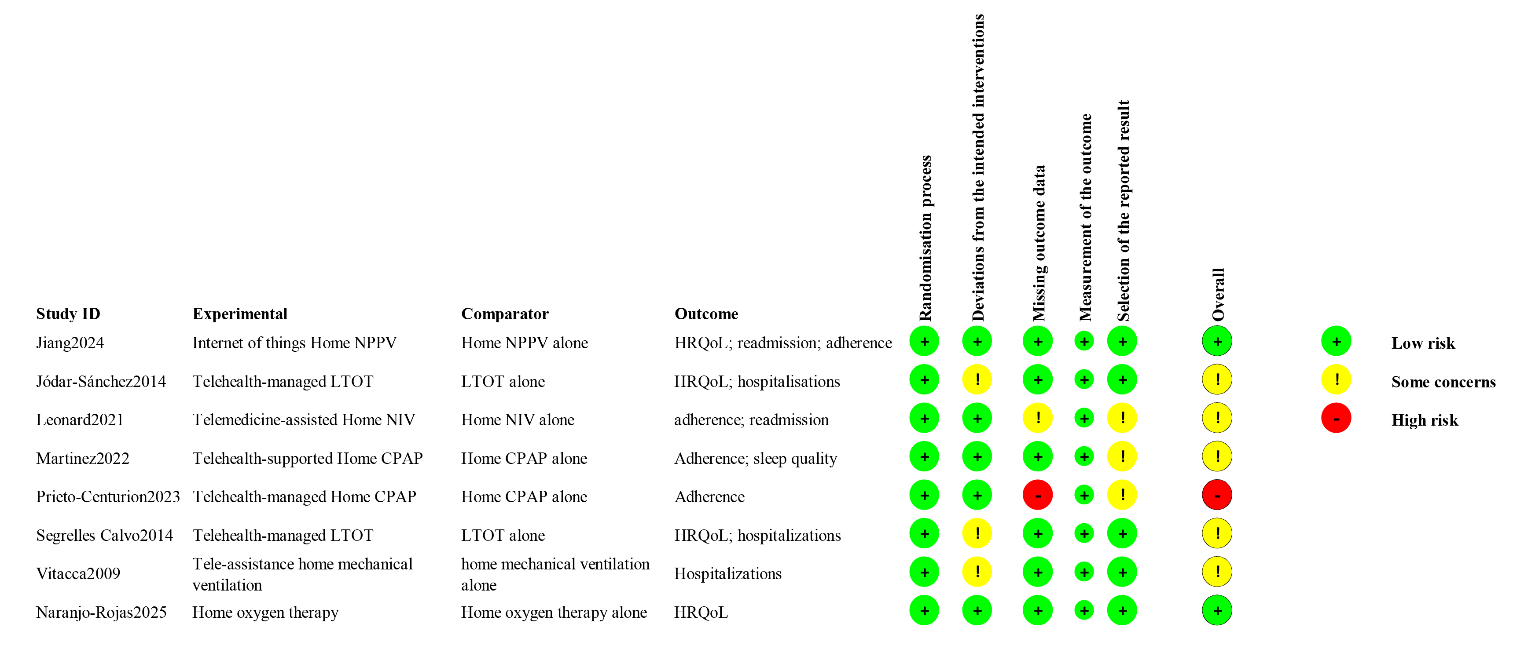


**Figure S4.** Risk of bias of the included studies.

**Table S2.** GRADE summary of the quality of the evidence for adherence, health-related quality of life and readmission

| **Certainty assessment** | | | | | | **Effect size** | **Quality of evidence** |
| --- | --- | --- | --- | --- | --- | --- | --- |
| **№ of studies** | **Risk of bias** | **Inconsistency** | **Indirectness** | **Imprecision** | **Publication bias** | **Absolute (95% CI)** |  |
| **Adherence** | | | | | | | |
| 3 | not serious^a^ | serious^b^ | not serious | serious^c^ | undetected^g^ | SMD **0.19 SD higher**  (-0.76 lower to 1.14 higher) | 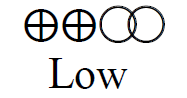 |
| **HRQOL** | | | | | | | |
| 4 | not serious^a^ | not serious^d^ | not serious | not serious^e^ | undetected^g^ | SMD **0.49 SD higher**  (0.25 higher to 0.73 higher) | 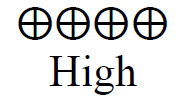 |
| **Hospital readmission** | | | | | | | |
| 3 | not serious^a^ | not serious^f^ | not serious | not serious^e^ | undetected^g^ | SMD -**0.40 SD lower**  (-0.60 lower to -0.21 higher) | 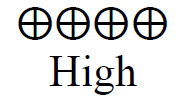 |

**Note:** CI: confidence interval; SMD: standardised mean difference; HRQOL: health-related quality of life.

**Explanations:**

a. Most of the information comes from studies rated as either low risk of bias or with some concerns.

b. An *I^2^* greater than 50% suggests substantial heterogeneity, likely attributable to differences in intervention targeting, treatment formats, and intervention durations.

c. The 95% confidence interval includes effect sizes that may not be clinically meaningful.

d. *I^2^* = 8.13%.

e. The 95% confidence interval excludes pooled effect sizes that are not clinically significant.

f. *I^2^* = 0.00%.

g. There is a lack of sufficient study (less than 10 studies) to detect publication bias.
